# The Nrf2-SLPI axis in aging and its role in the pathophysiology of pulmonary *Mycobacterium avium* complex disease

**DOI:** 10.3389/fimmu.2026.1733057

**Published:** 2026-02-25

**Authors:** Sosuke Matsumura, Masashi Matsuyama, Masayuki Nakajima, Chio Sakai, Kodai Ueda, Mizu Nonaka, Kengo Nishino, Zhenting Wei, Yuki Yabuuchi, Kenya Kuramoto, Kai Yazaki, Kazufumi Yoshida, Takumi Kiwamoto, Yuko Morishima, Yukio Ishii, Masafumi Muratani, Nobuyuki Hizawa

**Affiliations:** 1 Department of Pulmonary Medicine, Institute of Medicine, University of Tsukuba, Ibaraki, Japan; 2 Department of Pulmonary Medicine, National Hospital Organization Ibarakihigashi National Hospital, Ibaraki, Japan; 3 Department of Genome Biology, Faculty of Medicine, University of Tsukuba, Ibaraki, Japan

**Keywords:** aging, Nrf2, pulmonary *Mycobacterium avium* complex (MAC) disease, RNA-seq, secretory leukocyte protease inhibitor (SLPI)

## Abstract

Aging is associated with a poor prognosis in pulmonary *Mycobacterium avium* complex (MAC) disease. This study aimed to elucidate the impact of aging on pulmonary MAC disease and its underlying mechanisms. Young and old mice were intranasally infected with *Mycobacterium avium*. RNA-seq analysis was performed on lung tissues to identify age-related gene expression changes. Whole blood cells from 100 untreated patients with pulmonary MAC disease were analyzed for *SLPI* mRNA expression and its association with age and disease severity. Old mice were more susceptible to MAC infection than young mice, with increased bacterial load and decreased expression of secretory leukocyte protease inhibitor (SLPI) in the lungs. SLPI showed direct antimicrobial activity against *M. avium* and was regulated by Nrf2, a transcription factor with reduced activity in infected old mice. Nrf2-deficient mice showed decreased *SLPI* expression and increased bacterial load. Treatment with sulforaphane restored *SLPI* expression and reduced bacterial burden in old mice. In humans, cluster analysis identified three clusters based on age and *SLPI* expression. Compared to cluster 1 (C1) (younger age and high SLPI), cluster C3 (older age and lower SLPI) had larger pulmonary lesions on computed tomography. Pathway analysis indicated reduced Nrf2 activation in C3 than in C1, consistent with the findings in the mouse experiments. The study suggests that age-related reductions in Nrf2 activity and *SLPI* expression contribute to poor outcomes in pulmonary MAC disease. Targeting the Nrf2-SLPI axis may represent a novel therapeutic approach for elderly patients.

## Introduction

The incidence of pulmonary *Mycobacterium avium* complex (MAC) disease is increasing, and there is growing interest in this disease (1, 2), yet its pathophysiology remains poorly understood. Both host factors and environmental factors have been implicated in the pathogenesis of the disease (2). Of them, aging has emerged as a factor associated with a poor prognosis (3, 4). For example, the BACES score, which predicts mortality in pulmonary MAC disease, includes age ≥65 years as a key component (5). However, the mechanisms by which aging worsens prognosis remain unclear.

A previous study reported that old mice infected with *M. avium* have a reduced HO-1 response, increased bacterial burden, diffuse lung inflammation, impaired granuloma formation, and a decreased survival rate (6). Another report showed that aging was associated with increased disease severity and bacterial persistence in aged rhesus macaques (7). In addition, disease severity in aged rhesus macaques was mediated by a dysregulated macrophage response that may be sustained through persistent antigen presence (7). Despite these findings, the roles of the specific pathways affected have not been fully elucidated.

HO-1 is a well-known Nrf2 target gene (8), and Nrf2 activation has been reported to decline with aging (9, 10). Therefore, the reduced HO-1 response observed in aged mice (6) was suggested to result from decreased Nrf2 activity. Secretory leukocyte protease inhibitor (SLPI), expressed in airway epithelial cells, alveolar epithelium, and macrophages, protects against protease-mediated tissue damage, suppresses NF-κB–mediated inflammation, regulates NET formation, and has direct antimicrobial activity against bacteria, fungi, and *Mycobacterium tuberculosis*. However, the antibacterial activity of SLPI in NTM infection has not been elucidated. SLPI has also been reported to be regulated by Nrf2 (11–13). Therefore, a relationship between Nrf2 activation and SLPI expression in the lungs of MAC-infected old mice was also speculated.

In the present study, by examining MAC infection in young and old mice and analyzing gene expression in blood samples from patients with pulmonary MAC disease, the aim was to clarify the role of aging in disease pathogenesis, and it was shown that the age-related downregulation of the activity of the Nrf2-SLPI axis may be involved in the poor prognosis of elderly patients with pulmonary MAC disease.

## Methods

### Mycobacteria

*Mycobacterium avium* subsp. *hominissuis*, isolated from a patient with pulmonary MAC disease, was used as the *M. avium* complex (MAC) bacterium, the same bacteria used in the previous report (14). The bacterium was grown to mid-log phase in Middlebrook 7H9 liquid medium (Difco/Becton Dickinson), aliquoted, and frozen at −80 °C until use. Bacterial counts in each organ were determined by plating serial dilutions of organ homogenates from individual mice on Middlebrook 7H10 agar plates and counting bacterial colonies (colony forming unit, CFU) 2 weeks after plating.

### Mice and infection

Wild-type BALB/c mice were purchased from The Jackson Laboratory Japan (Yokohama, Japan). *Nrf2^−/−^* mice were generated as described (15) and backcrossed with BALB/c mice for nine generations. Female wild-type mice (8 to 12 weeks old [young mice] and 18 months old [old mice]) and female Nrf2-deficient mice (8 to 12 weeks old) were infected with MAC by intranasal inoculation at a dose of 1 × 10^7^ CFU in 50 μL of phosphate-buffered saline (PBS). Control mice were treated with 50 μL of PBS. After anesthesia with isoflurane, the MAC bacterial suspension or PBS was administered repeatedly intranasally, one drop at a time. Wild-type BALB/c mice and Nrf2-deficient mice were mated and bred in the specific pathogen-free (SPF) facility (Laboratory Animal Resource Center in University of Tsukuba). Female mice were raised 5–7 mice per cage. Infection was performed at P2 level infection laboratory of Laboratory Animal Resource Center in University of Tsukuba. All animal studies were approved by the Institutional Review Board in University of Tsukuba (approval number 24-269).

### Histology

Lung sections were stained with hematoxylin and eosin. Ziehl-Neelsen staining was used to detect bacilli. Paraffin-embedded sections of lung tissue were immunostained with an antibody against SLPI (NOVUS: NBP1-76803) using the universal immuno-enzyme polymer method (Histofine Simple Stain; Nichirei, Tokyo, Japan). For immunofluorescence staining, lung sections were incubated with appropriately diluted primary antibodies (SLPI [NOVUS: NBP1-76803] and F4/80 [Cell Signaling TECHNOLOGY: #70076S]). Secondary antibodies were species-specific, matching the source of the primary antibodies (SLPI [Alexa Fluor 488] and F4/80 [Alexa Fluor 594]). Finally, the sections were counterstained with DAPI (VECTASHIELD MOUNTING MEDIUM). The stained sections were then observed, with a fluorescence microscope, OLYMPUS DP70 (OLYMPUS, Japan).

### Bronchoalveolar lavage

The lungs were lavaged with six sequential 1-mL aliquots of saline. Cells were counted using a hemocytometer, and differential cell counts were obtained by staining with Diff-Quick (Polysciences, Inc.) after cytospins.

### Reverse transcription-PCR

Total RNA was extracted from lungs using the RNeasy mini kit (Qiagen). Real-time quantitative reverse transcription-PCR (RT-PCR) was performed using QuantStudio 5 (Applied Biosystems). The PCR primers used in this study are listed in **Supplementary Table 1**. Target gene expression levels were calculated using the ΔΔCT method and normalized against glyceraldehyde 3-phosphate dehydrogenase mRNA.

### Antimicrobial activity of SLPI

Mouse SLPI/anti-leukoproteinase recombinant (His+S) protein (aa20-131) (LifeSpan Biosciences, Inc.) was used. The SLPI protein was dissolved in PBS. The MAC suspension was diluted with 7H9 liquid. *M. avium* (2.0 × 10^6^ CFU/ml) was incubated with SLPI (3 μM) or PBS as a control at 37 °C for 7 days. Colonies were counted (CFU/ml) at 24 h, 3 days, and 7 days.

### Western blot analysis

Nuclear extracts were prepared from lung tissue using a nuclear extraction kit (Invent Biotechnologies, Inc., Plymouth City, MN, USA), according to the manufacturer’s instructions. Ten to twenty micrograms of nuclear extracts were separated with 10% SDS-PAGE gels and transferred to polyvinylidene difluoride (PVDF) membranes. After blocking for nonspecific sites, the PVDF membranes were incubated with anti-Nrf2 antibody (D1Z9C), followed by incubation with secondary antibodies (IRDye^®^ 680RD donkey anti-rabbit IgG; LICOR bio). Immunoreactive bands were visualized using a near-infrared fluorescence imaging system (ODYSSEY CLx, LICOR bio). Lamin B was used as an internal control. Values were normalized to lamin B to evaluate Nrf2 expression.

### SFN treatment

R, S-sulforaphane (SFN; LKT Laboratories Inc., St. Paul, MN, USA) was used in this study. A stock solution of SFN was prepared using ethanol as the solvent and stored at −20 °C in the dark. The SFN stock solution was diluted with PBS immediately before use. SFN was injected intraperitoneally into 18-month-old mice at a dose of 5 mg/kg, 5 days/week for 4 weeks immediately after intranasal inoculation of MAC.

### RNA-seq analysis of mouse lung tissue

Total RNA was extracted from lung tissues using TRIzol with a homogenizer in 12 individual samples (3 from the lungs of the young mice treated with PBS, 3 from the lungs of the old mice treated with PBS, 3 from the lungs of the young mice infected with *M. avium* for 2 months, and 3 from the lungs of the old mice infected with *M. avium* for 2 months); 500  ng of total RNA were used for RNA-seq. Sequencing was performed with NextSeq500 (Illumina) by Tsukuba i-Laboratory LLP (Tsukuba, Ibaraki, Japan). FASTQ files were analyzed using CLC Genomics Workbench (CLC-GW, version 10.1.1; Qiagen). Reads were mapped to the mouse reference genome (mm10) and quantified for annotated genes. The Empirical Analysis of DGE tool in CLC-GW was used to detect differential expression of genes (FDR- adjusted P<0.001 and with more than 2-fold changes). The data are available under GEO series accession number GSE287501. Heat maps were generated using Morpheus (https://software.broadinstitute.org/morpheus/), and Venn diagrams were generated using Venny 2.1.0 (https://bioinfogp.cnb.csic.es/tools/venny/).

### RNA-seq analysis of human whole blood cells

A multicenter, non-interventional, prospective, observational study was performed. The study protocol was approved by the ethics committees of Tsukuba University Hospital and other hospitals, and the study was performed in accordance with the Declaration of Helsinki (approval number R01-379). Written, informed consent was obtained from all participating patients. Between May 2020 and December 2024, 100 patients consented to participate after being screened for eligibility.

The participants were 100 patients newly diagnosed with pulmonary MAC disease according to ATS/ERS/ESCMID/IDSA criteria and scheduled to receive guideline-based therapy (GBT) at the discretion of their treating physicians. Baseline characteristics prior to GBT were obtained from patients’ medical records, including age, sex, body mass index (BMI), smoking status, comorbidities, presence of respiratory symptoms, St. George’s Respiratory Questionnaire (SGRQ) score, and laboratory test results. Laboratory data were collected, including white blood cell count, neutrophil count, lymphocyte count, monocyte count, eosinophil count, basophil count, albumin, CRP, and GPL-core IgA antibody. A GPL core IgA antibody titer ≥0.7 U/mL was considered positive. For radiological evaluation, the locations of the lesions and the number of lobes involved were analyzed. The locations of the lesions were evaluated in separate sections: upper, middle (lingula), and lower lobes, with the lingula considered a separate lobe. The number of lobes with lesions was defined as the computed tomography (CT) score. As for the BACES score, a CRP of 0.3 or higher (1 point) was used as a substitute for an elevated erythrocyte sedimentation rate (ESR) (1 point), since ESR was not measured in this study. This new score was called the BACScrp score instead of the BACES score. Sputum samples were inoculated into MGIT liquid media and Ogawa solid media.

Blood was collected from all participants before treatment using PAXgene blood RNA tubes (Qiagen). Silica membrane-based RNA isolation and purification in a spin-column format were performed using the PAXgene Blood RNA kit (Qiagen). Sequencing was performed by Tsukuba i-Laboratory LLP using NextSeq500 (Illumina) for whole blood RNA. Sequencing reads were mapped to the hg19 human reference genome and quantified using CLC Genomics Workbench version 10.1.1 (Qiagen). Differentially expressed genes were identified by filtering according to the P-values obtained by Empirical Analysis of DGE tool in CLC-GW. Genes were identified as differentially expressed if they had an adjusted (Benjamini-Hochberg FDR method for correction of multiple testing) p-value of 0.05. Data are available under the GEO series accession number GSE 288604.

### Statistical analysis

#### Analysis of the data from mice

Data are expressed as mean and standard error of the mean (SEM) values. Student’s *t*-test was used to compare data between two groups. One-way analysis of variance (ANOVA) followed by the *post hoc* Tukey’s test was used for comparative analysis among three or more groups. Survival data were analyzed using the Kaplan-Meier method and the log-rank test. P values of ≤0.05 were considered significant. IBM SPSS Statistics 29.0 (IBM Corp, Armonk, NY, USA) and GraphPad Prism version 7 (Graph Pad Software Inc.) were used for statistical analysis.

#### Analysis of the data from patients with pulmonary MAC disease

Categorical variables are described by number (n), and continuous variables are described by mean ± standard deviation (SD) or median (interquartile range) values. The chi-squared test was performed for comparative analysis of categorical variables between each cluster. For comparative analysis of continuous variables, one-way analysis of variance (ANOVA) followed by the *post hoc* Tukey’s test was performed if the data were found to be normally distributed; otherwise, the Kruskal-Wallis test followed by the *post hoc* Dunn’s test was used. A two-step cluster analysis was performed to identify distinct clusters among the 100 patients with pulmonary MAC disease. The input variables were age and secretory leukocyte protease inhibitor (*SLPI*) mRNA expression levels. The two-step cluster analysis in SPSS is a method used to identify natural clusters. This approach allows for the automatic determination of the optimal number of clusters by incorporating both categorical and continuous variables. The analysis is conducted in two stages. In the first stage, a preliminary clustering process is performed using the log-likelihood distance, resulting in the creation of small sub-clusters. In the second stage, these sub-clusters are hierarchically merged to form the final clusters. The optimal number of clusters is determined based on Schwarz’s Bayesian Information Criterion (BIC). Upstream regulator analysis, which provides predictions of activation or inhibition of upstream regulators based on gene expression and a previous study database (https://digitalinsights.qiagen.com/products-overview/discovery-insights-portfolio/analysis-and-visualization/qiagen-ipa/features/upstream-regulator-analysis/), was performed by IPA (Qiagen). CIBERSORT x (https://cibersortx.stanford.edu/) was performed with LM22, which is a signature matrix file consisting of genes that distinguish 22 mature human hematopoietic cells. P values of ≤0.05 were considered significant. IBM SPSS Statistics 29.0 (IBM Corp) and GraphPad Prism version 7 (Graph Pad Software Inc.) were used for statistical analysis.

## Results

### Susceptibility to MAC is higher in infected old mice than in infected young mice

**Figure 1:**
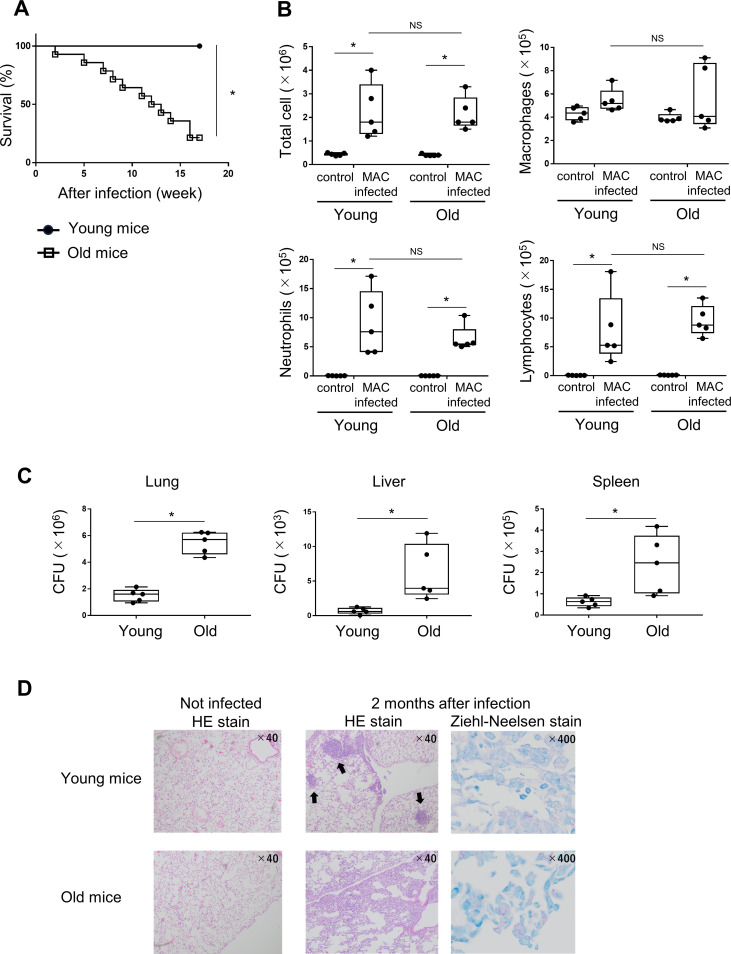
Survival rate, bronchoalveolar lavage fluid (BALF), organ bacterial load, and lung pathology of young and old mice after MAC infection. **(A)** Survival of young and old mice after intranasal inoculation of 1×10^7^ colony-forming units (CFU) of MAC bacteria (n = 14). Experiments were performed in duplicate. **(B)** The numbers of total cells, neutrophils, macrophages, and lymphocytes from BALF of young and old mice 2 months after intranasal inoculation of 1×10^7^ CFU of MAC or PBS (Cont). All experiments were performed in duplicate with five mice per group. **(C)** Mycobacterial outgrowths in the lung, spleen, and liver of young and old mice 2 months after intranasal inoculation of 1×10^7^ CFU of MAC. Results are expressed as CFU per organ. Experiments were performed in duplicate with five mice in each group. **(D)** Representative photomicrographs of the lungs of young mice and old mice 2 months after intranasal inoculation of 1×10^7^ CFU of MAC. The organized granulomas are indicated by closed arrows. Hematoxylin and eosin staining (×40) and Ziehl-Neelsen staining (×400) were used. *Significant difference between each group (p<0.05). Data are expressed as mean ± SEM values.

To determine the effect of aging on MAC infection in mice, the survival rate, bronchoalveolar lavage fluid (BALF), organ bacterial load, and lung pathology of mice 2 months after MAC infection were examined. The survival rate after infection was significantly lower in old mice than in young mice (**Figure 1A**). The number of inflammatory cells in BALF was not significantly different between young and old mice 2 months after MAC infection (**Figure 1B**). Organ colony-forming unit (CFU) measurement showed higher mycobacterial counts in lungs, livers, and spleens of old mice than in young mice 2 months after MAC infection (**Figure 1C**). Lung pathology 2 months after infection was evaluated by HE staining. Both young and old infected mice showed inflammatory cell infiltration in the lungs, but granuloma formation was poor in infected old mice (**Figure 1D**). These results suggest that old mice are more susceptible to MAC infection than young mice.

### The expression of SLPI in the lung tissue is attenuated in MAC-infected old mice compared with MAC infected young mice

**Figure 2:**
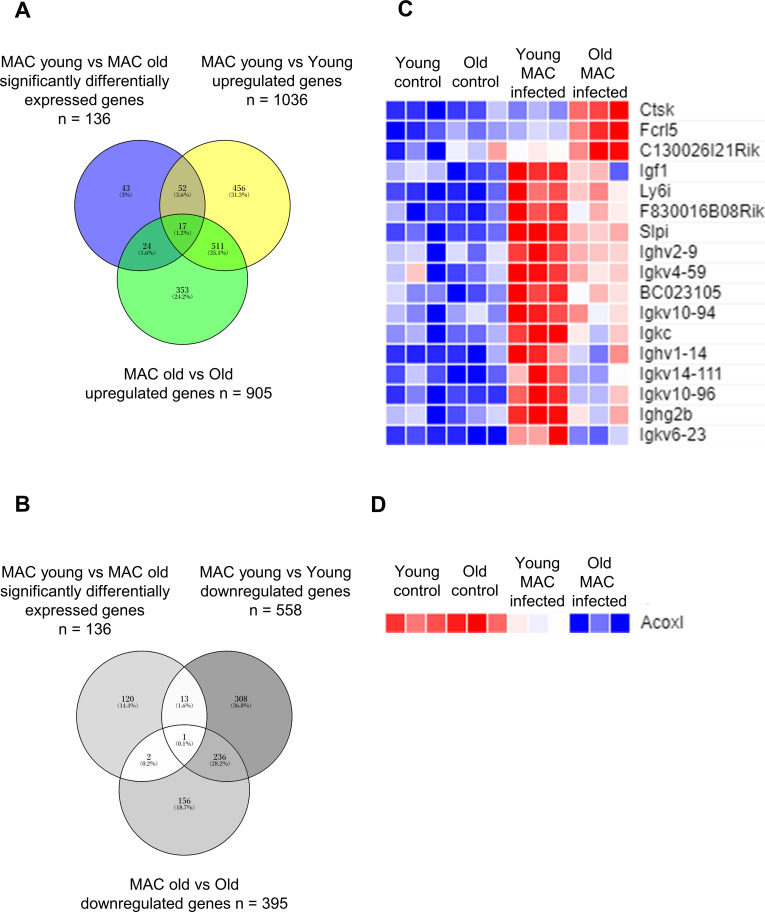
Comprehensive gene expression analysis in lung tissues of young and old mice 2 months after MAC infection. Overlap comparison of differentially expressed genes (DEGs) detected in each DEG analysis, **(A)** Venn diagram of DEGs between MAC-infected young mice and MAC-infected old mice (136 genes), upregulated DEGs between MAC-infected young mice and uninfected young mice (1036 genes), and upregulated DEGs between MAC-infected old mice and uninfected old mice (905 genes). **(B)** Venn diagram of DEGs between MAC-infected young mice and MAC-infected old mice (136 genes), downregulated DEGs between MAC-infected young mice and uninfected young mice (558 genes), and downregulated DEGs between MAC-infected old mice and uninfected old mice (395 genes). **(C)** The heat map based on transcripts per million (TPM) of 17 genes that were upregulated after infection in both young and old mice, and whose expression levels differ significantly between infected young mice and infected old mice. **(D)** The heat map based on TPM of one gene that was downregulated after infection in both young and old mice, and whose expression level is significantly different between infected young mice and infected old mice.

To understand the detailed mechanisms explaining why old mice have high susceptibility to MAC infection, RNA-seq of lung tissues was performed using young and old mice with or without MAC infection. The mRNA expression levels were compared and analyzed between uninfected and infected young lungs, between uninfected and infected old lungs, and between infected young lungs and infected old lungs (FDR-adjusted P<0.001 and with more than 2-fold changes) (**Supplementary Table 2**; **Figures 2A, B**). There were 17 genes that were upregulated in both young and old mice 2 months after infection, and their expression levels were significantly different between infected young mice and infected old mice (**Figures 2A, C**). In addition, there was one gene that was downregulated in both young and old mice 2 months after infection, and its expression level was significantly different between infected young and infected old mice (**Figures 2B, D**). Of these 18 genes, secretory leukocyte protease inhibitor (SLPI), which encodes a peptide with antimicrobial and antiprotease activity, was selected for evaluation as a potential gene involved in the control of MAC infection.

### SLPI is expressed on macrophages in MAC-infected lung tissue

**Figure 3:**
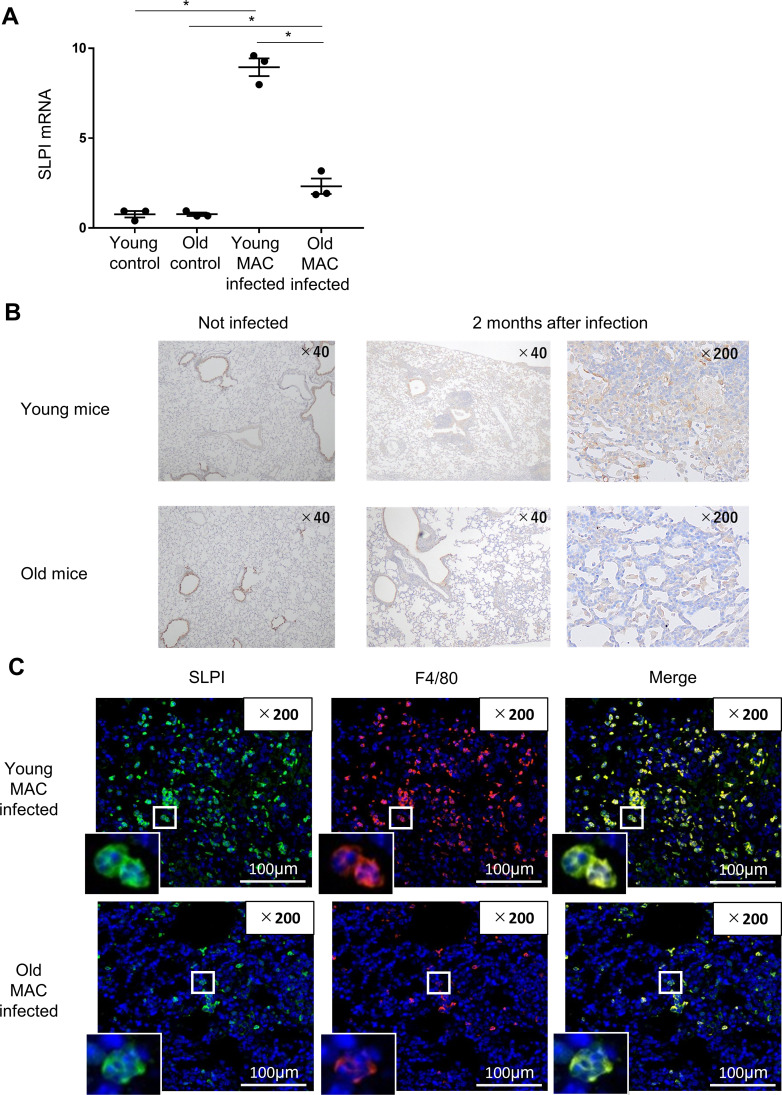
Localization of SLPI. **(A)** Validation of RNA-seq data by performing RT-qPCR for SLPI in lung tissues 2 months after MAC infection. Experiments were performed in duplicate with three mice per group. **(B)** Representative photographs of immunostaining for SLPI in the lungs of young and old mice 2 months after MAC infection (×40 and ×200). **(C)** Representative images showing SLPI expression in the lungs of young and old mice 2 months after MAC infection (immunofluorescence, SLPI in green, F4/80 in red, and DAPI in blue) (×200). Significant difference between each group (p < 0.05). Data are expressed as mean ± SEM values.

To validate the RNA-seq data, *SLPI* mRNA expression was assessed by qPCR in lung tissues from young and old mice infected with MAC bacteria. Similar to the RNA-seq result, the expression of *SLPI* was induced in both mice after MAC infection, but it was significantly decreased in infected old mice compared to infected young mice (**Figure 3A**). To confirm the expression and localization of SLPI protein in MAC-infected lung tissues, SLPI immunostaining was performed using anti-SLPI antibody. SLPI was expressed in the bronchial epithelial cells of both young and old mice at the same level before infection. In the infected lung tissue, there was no change in the expression of SLPI protein in bronchial epithelial cells. However, SLPI expression was observed in inflammatory cells, mainly macrophages, in both mice and was higher in young mice than in old mice (**Figures 3B, C**).

### SLPI has antimicrobial activity against MAC bacteria

**Figure 4:**
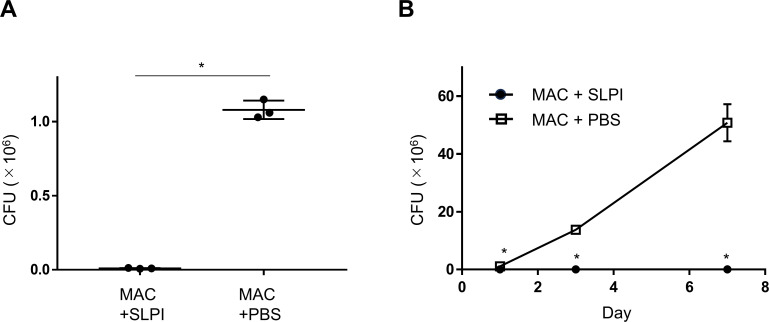
Antibacterial activity of SLPI against *M. avium.* **(A, B)** *In vitro* effect of SLPI on *M. avium*. **(A)** *M. avium* was incubated with SLPI or PBS as a control at 37 °C for 24 h, and the amount of MAC bacteria was evaluated. Experiments were performed in duplicate with three technical replicates in each group. **(B)** *M. avium* was incubated with SLPI or PBS as a control at 37 °C for 7 days, and mycobacterial outgrowth was evaluated on day 1, day 3, and day 7. Experiments were performed in duplicate with three technical replicates in each group. *Significant difference between each group (p<0.05). Data are expressed as mean ± SEM values.

To examine the antibacterial effect of SLPI on MAC bacteria, SLPI protein was added directly to MAC bacteria. After 24 hours of incubation, the SLPI-treated group showed a significant reduction in the amount of MAC bacteria compared with the control group, to almost zero CFU (**Figure 4A**). After 3 and 7 days of incubation, the SLPI-treated group showed no increase in the number of bacteria throughout the observation period, whereas the control group showed an increase in the number of MAC bacteria (**Figure 4B**). These results suggest that SLPI itself has an antibacterial effect on MAC bacteria.

### Nrf2 activity is decreased in the lungs of old mice compared with young mice

**Figure 5:**
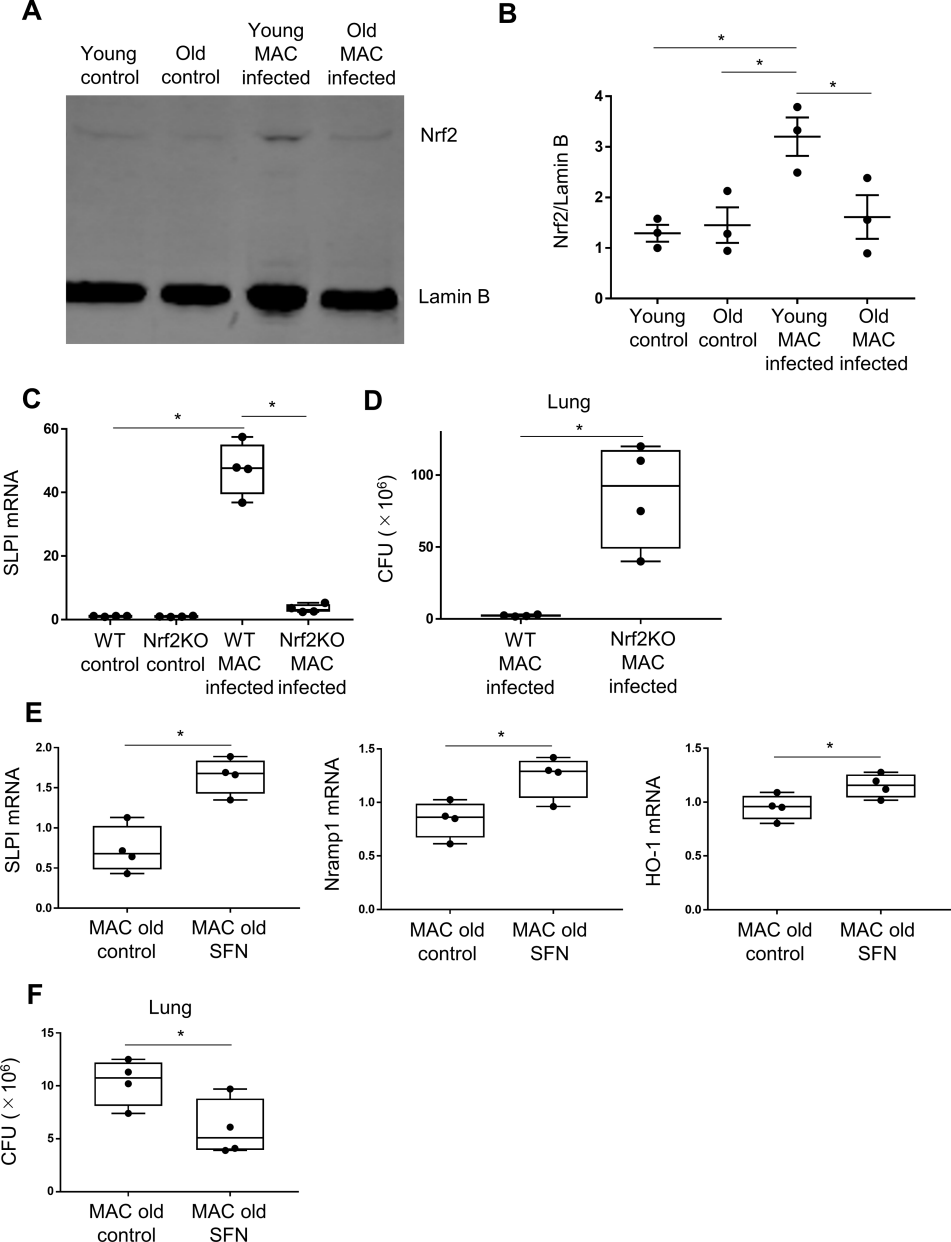
Relationship between SLPI and Nrf2. **(A, B)** Representative Western blots of SLPI expression **(A)** and its semiquantitative analysis **(B)** in lung nuclear extracts from young mice and old mice, infected or non-infected with MAC bacteria. Values were normalized to lamin **(B)** Experiments were performed in duplicate with three mice in each group. **(C)** Expression of SLPI was evaluated in infected lungs of wild-type and Nrf2-deficient mice. Experiments were performed in duplicate with four mice per group. **(D)** Mycobacterial outgrowth in the lungs of wild-type and Nrf2^−/−^ mice 2 months after intranasal inoculation of 1 × 10^7^ CFU of MAC bacteria. Results are expressed as CFU per organ. Experiments were performed in duplicate with four mice per group. **(E)** Expression of SLPI, Nramp-1, and HO-1 by RT-qPCR in the lungs of old mice 1 month after intranasal inoculation of 1 × 10^7^ CFU of MAC with or without sulforaphane treatment. Experiments were performed in duplicate with four mice per group. **(F)** Mycobacterial outgrowths in the lungs of old mice 1 month after intranasal inoculation of 1 × 10^7^ CFU of MAC, with or without treatment with sulforaphane. Results are expressed as CFU per organ. Experiments were performed in duplicate with four mice in each group. *Significant difference between each group (p<0.05). Data are expressed as mean ± SEM values.

SLPI has been reported to be regulated by Nrf2 (11–13). It has also been reported that Nrf2 activation decreases with aging (9, 10). Therefore, a relationship between Nrf2 activation and SLPI expression in the lungs of MAC-infected old mice was hypothesized, and whether nuclear translocation of Nrf2 occurs in the lung tissues after MAC infection was investigated. Western blot analysis showed that the degree of nuclear translocation of Nrf2 was higher in the infected young lungs than in the infected old lungs (**Figures 5A, B**). These results indicated that a significant decrease in Nrf2 activation was observed in the lungs of infected old mice compared with infected young mice.

### In *Nrf2*-deficient mice, *SLPI* expression in the lung is significantly reduced during MAC infection, and the number of bacteria is greatly increased

Next, to confirm that *SLPI* expression in the lung is regulated by Nrf2 during MAC infection, BALB/c *Nrf2*-deficient mice were infected intranasally with MAC bacteria, and *SLPI* expression and bacterial load in the lungs 2 months after infection were examined. *SLPI* expression was significantly lower in the lungs of *Nrf2*^-/-^ mice than in those of wild-type mice (**Figure 5C**). In addition, organ CFU measurement showed increased mycobacterial counts in the lungs of *Nrf2*^-/-^ mice compared with wild-type mice 2 months after MAC infection (**Figure 5D**). These results suggest that *SLPI* expression is regulated by Nrf2 and may contribute to infection control in MAC infection.

### SFN stimulation increases Nrf2 activity, induces *SLPI* expression, and reduces bacterial load

To confirm whether activation of Nrf2 induces *SLPI* expression and reduces the growth of MAC bacteria, old mice were treated with SFN during MAC infection. SFN increased the expression of *SLPI* (**Figure 5E**). The number of mycobacteria in the lungs was also reduced after SFN treatment (**Figure 5F**). Thus, it was confirmed that SFN suppressed the growth of MAC correlating with increased lung expression of *SLPI* and other Nrf2-associated genes in old mice.

### Patients with pulmonary MAC disease were classified into three clusters by age and mRNA expression of *SLPI* in whole blood cells

**Figure 6:**
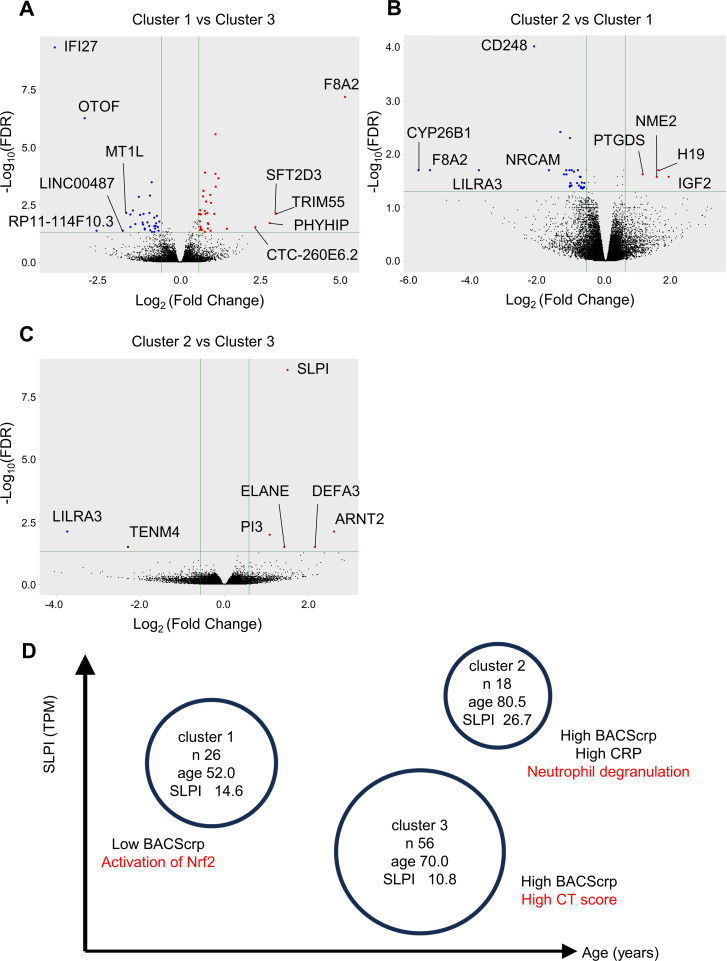
Two-step cluster analysis using age and TPM levels of SLPI in whole blood cells from 100 patients with pulmonary MAC disease. **(A-C)** Volcano plot of differentially expressed genes between each cluster. The top 5 downregulated or upregulated genes are listed. Blue dots represent downregulated genes, and red dots represent upregulated genes. **(A)** Volcano plot of differentially expressed genes between cluster C1 and C3. **(B)** Volcano plot between C2 and C1. **(C)** Volcano plot between C2 and C3. **(D)** A summary of the phenotypes identified by cluster analysis in 100 patients with pulmonary MAC disease. The clusters are plotted according to their relative expression of TPM levels of SLPI and age. The size of the circle indicates the sample size. TPM, transcripts per million; SLPI, secretory leukocyte protease inhibitor.

Experiments in old mice suggested that decreased Nrf2-regulated *SLPI* expression by macrophages in MAC-infected lungs is associated with the poor prognosis with age. We hypothesized that, in humans as well, age-related decreases in SLPI expression may contribute to the worsening of pulmonary MAC disease. To investigate the relationships of *SLPI* expression and age with disease severity in humans, a two-step cluster analysis using patient age and *SLPI* mRNA expression levels in whole blood cells from 100 patients with pulmonary MAC disease was performed. These 100 patients were newly diagnosed and scheduled to receive GBT at the discretion of their treating physicians. The analysis identified three clusters (**Table 1**): Cluster 1 (C1), younger patients (median age: 52.0 years) with moderate *SLPI* expression (median transcripts per million [TPM]: 14.6, 26 cases); Cluster 2 (C2), older patients (median age: 80.5 years) with high *SLPI* expression (median TPM: 26.7, 18 cases); and Cluster 3 (C3), predominantly older patients (median age: 70.0 years) with low *SLPI* expression (median TPM: 10.3, 56 cases). Compared to C1, C3 had larger pulmonary lesions on computed tomography (CT). Differentially expressed gene (DEG) analysis showed 88 DEGs between C1 and C3, 46 DEGs between C1 and C2, and 7 DEGs between C2 and C3 (FDR < 0.05) (**Figures 6A-C**; **Supplementary Table 3**). Upstream regulator analysis in IPA (Qiagen) showed reduced Nrf2 activation in C3 compared with C1, consistent with the findings from the mouse experiments (**Supplementary Figure 1**; **Supplementary Table 4**). These characteristics of C3 closely resembled those observed in MAC-infected old mice, suggesting that reduced *SLPI* expression may contribute to human pulmonary MAC disease in the context of aging, which is associated with a poor prognosis. Although C2 was also an older group, it was characterized by high *SLPI* expression (median TPM: 26.7). The CRP level in C2 was higher than in both C1 and C3, and the BACScrp score in C2 was significantly higher than in C1 (**Figure 6D**). Data from CIBERSORTx, a program that estimates the proportion of immune cells in each cluster based on gene expression profiles, showed a higher proportion of neutrophils in C2 (**Table 2**; **Supplementary Figure 2**). In addition, expression of neutrophil-related genes, such as *DEFA3* and *ELANE*, was significantly higher in C2 than in C3. These findings suggest that C2 represents a group of more severe cases in which neutrophil-driven inflammation plays a significant role (**Figure 6D**).

**Table 1:** Clinical characteristics of patients with pulmonary Mycobacterium avium complex disease classified by cluster analysis.

Variable	N= 100	Cluster 1 (n= 26)	Cluster 2 (n= 18)	Cluster 3 (n= 56)	*P* value
SLPI	13.7 (8.3 - 22.2)	14.6 (8.3 – 24.5) ^a,c^	26.7 (26.0 - 34.0) ^a,b^	10.3 (7.0 – 14.3) ^b,c^	**<0.001**
Age (y)	68.0 (60.0 - 77.0)	52.0 (48.0 – 56.0) ^a,c^	80.5 (72.0 – 84.0) ^a,b^	70.0 (66.0 - 77.0) ^b,c^	**<0.001**
Sex Male/Female, n	29/71	4/22	7/11	18/38	0.177
BMI (kg/m^2^)	19.7 ± 2.8	20.3 ± 2.6	19.6 ± 3.3	19.5 ± 2.8	0.543
Smoking (never/former/current)	67/27/6	16/7/3	11/7/0	40/13/3	0.398
Pack years	0.0 (0.0 - 10.6)	0.0 (0.0 - 10.0)	0.0 (0.0 - 13.0)	0.0 (0.0 - 10.6)	0.771
Anti-MAC antibodies positive, n (%)	83 (83.0)	22 (84.6)	16 (88.9)	45 (80.4)	0.681
BACScrp	2.0 (1.0 - 3.0)	1.0 (0.0 - 1.0) ^a,c^	3.0 (1.0 - 3.0) ^a^	2.0 (1.0 - 3.0) ^c^	**<0.001**
Sputum smear positive, n (%)	13 (13.0)	3 (11.5)	2 (11.1)	8 (14.3)	0.910
Sputum culture positive, n (%)	73 (73.0)	18 (69.2)	17 (94.4)	38 (67.9)	0.077
SGRQ score	11.8 (5.7 - 27.9)	14.0 (4.1 - 29.9)	18.9 (11.1 – 42.9)	11.0 (5.3 - 24.9)	0.124
Cavity, n (%)	32 (32.0)	7 (26.9)	8 (44.4)	17 (30.4)	0.436
CT score	4.0 (3.0 - 5.5)	3.0 (3.0 – 4.0) ^c^	5.0 (3.0 - 6.0)	4.0 (3.0 - 6.0) ^c^	**0.041**
FEV1 (ml)	1760.0 (1520.0 - 2180.0)	2115.0 (1900.0 - 2400.0) ^a,c^	1620.0 (1400.0 – 1950.0) ^a^	1670.0 (1480.0 - 1980.0) ^c^	**<0.001**
FVC (ml)	2240.0 (1880.0 - 2700.0)	2660.0 (2380.0 – 3100.0) ^a,c^	2080.0 (1700.0 – 2600.0) ^a^	2070.0 (1800.0 – 2510.0) ^c^	**<0.001**
FEV1% (%)	79.7 (75.2 - 86.0)	78.8 (74.8 – 83.6)	79.5 (77.7 – 81.9)	81.1 (75.5 - 87.4)	0.361
CRP (mg/dl)	0.06 (0.03 - 0.21)	0.055 (0.030 - 0.100) ^a^	0.175 (0.060 - 1.250) ^a,b^	0.055 (0.030 - 0.225) ^b^	**0.027**
Alb (g/dl)	4.10 (3.80 – 4.35)	4.30 (4.00 - 4.45) ^a^	3.90 (3.60 – 4.20) ^a^	4.10 (3.85 – 4.35)	**0.035**

Definitions of abbreviations: SLPI, secretory leukocyte protease inhibitor; BMI, body mass index; MAC, *Mycobacterium avium* complex; SGRQ, st. george’s respiratory questionnaire; FEV1, forced expiratory volume in one second; FVC, forced vital capacity; FEV1%, forced expiratory volume % in one second; CRP, C reactive protein; Alb, albumin. Data are presented as n, n (%), means ± standard deviation, or medians (interquartile range). *P* values are for comparisons between cluster 1, cluster 2, and cluster 3. Categorical variables were analyzed using the Chi-squared test. Continuous variables with normal distributions are shown as means ± standard deviation. Continuous variables with non-normal distribution are shown as medians and interquartile range. Non-normally distributed variables were analyzed by the Kruskal-Wallis followed by a Dunn’s *post hoc* comparison, whereas normally distributed parameters were assessed by the one-way ANOVA followed by a Tukey’s *post hoc* comparison. Bold indicates significance (*p* < 0.05). a; cluster 1 *vs* cluster 2 (p<0.05). b; cluster 2 *vs* cluster 3 (p<0.05). c; cluster 1 *vs* cluster 3 (p<0.05).

**Table 2:** Estimated immune cell proportions in each cluster based on CIBERSORTx analysis.

Variable	N = 100	Cluster 1 (n= 26)	Cluster 2 (n= 18)	Cluster 3 (n= 56)	*P* value
B cells naive	2.2 (0.9 - 4.7)	3.5 (1.7 - 6.1) ^a^	1.3 (0.6 - 1.9) ^a^	2.5 (0.6 - 5.2)	**0.005**
B cells memory	0.0 (0.0 - 0.0)	0.0 (0.0 - 0.0)	0.0 (0.0 - 0.0)	0.0 (0.0 - 0.1)	0.947
Plasma cells	0.35 (0.02 - 0.84)	0.2 (0.0 - 0.5) ^a^	0.7 (0.3 - 1.0) ^a,b^	0.3 (0.0 - 0.8) ^b^	**0.023**
T cells CD8	8.6 ± 4.4	8.7 ± 3.8	7.4 ± 3.0	8.9 ± 4.9	0.429
T cells CD4 naive	2.1 (0.0 - 4.6)	3.1 (0.6 - 5.9) ^a^	1.5 (0.0 - 2.6) ^a^	2.0 (0.0 - 4.6)	**0.017**
T cells CD4 memory resting	6.1 (3.8 - 9.3)	8.0 (3.6 - 10.9)	5.4 (2.6 - 6.3)	6.1 (4.0 - 9.3)	0.059
T cells CD4 memory activated	0.89 (0.04 - 1.9)	1.3 (0.04 - 1.9)	1.7 (0.6 - 2.0) ^b^	0.5 (0.0 - 1.5) ^b^	**0.032**
T cells follicular helper	0.0	0.0	0.0	0.0	–
T cells regulatory	0.21 (0.00 - 1.2)	0.2 (0.0 - 1.2)	0.0 (0.0 - 0.8)	0.3 (0.0 - 1.2)	0.448
T cells gamma delta	0.0 (0.0 - 0.0)	0.0 (0.0 - 0.0)	0.0 (0.0 - 0.0)	0.0 (0.0 - 0.0)	0.241
NK cells resting	10.3 (7.2 - 14.2)	7.8 (5.7 - 11.4) ^c^	10.5 (8.1 - 11.6)	11.3 (8.1 - 15.9) ^c^	**0.036**
NK cells activated	0.0 (0.0 - 0.0)	0.0 (0.0 - 0.0)	0.0 (0.0 - 0.0)	0.0 (0.0 - 0.0)	0.596
Monocytes	15.3 (11.4 - 17.8)	14.7 (11.0 - 17.7)	15.6 (11.8 - 17.0)	15.3 (11.4 - 19.0)	0.894
Macrophages M0	0.0 (0.0 - 0.0)	0.0 (0.0 - 0.0)	0.0 (0.0 - 0.0)	0.0 (0.0 - 0.0)	0.456
Macrophages M1	0.0	0.0	0.0	0.0	-
Macrophages M2	0.0	0.0	0.0	0.0	–
Dendritic cells resting	0.0	0.0	0.0	0.0	-
Dendritic cells activated	0.06 (0.00 - 0.3)	0.1 (0.0 - 0.6)	0.1 (0.0 - 0.5)	0.0 (0.0 - 0.3)	0.413
Mast cells resting	1.5 (1.1 - 2.2)	2.0 (1.0 - 2.2)	1.9 (1.1 - 2.7)	1.4 (0.9 - 1.9)	0.146
Mast cells activated	0.0	0.0	0.0	0.0	–
Eosinophils	0.0 (0.0 - 0.0)	0.0 (0.0 - 0.0)	0.0 (0.0 - 0.0)	0.0 (0.0 - 0.0)	0.675
Neutrophils	48.5 ± 12.1	48.3 (44.2 - 56.0)	56.7 (54.3 - 58.9) ^b^	47.5 (35.7 - 54.6) ^b^	**0.017**

Definitions of abbreviations: NK, natural killer. Data are presented as means ± standard deviation or medians (interquartile range). *P* values are for comparisons between cluster 1, cluster 2, and cluster 3. Continuous variables with normal distributions are shown as means ± standard deviation. Continuous variables with non-normal distribution are shown as medians and interquartile range. Non-normally distributed variables were analyzed by the Kruskal-Wallis followed by a Dunn’s *post hoc* comparison, whereas normally distributed parameters were assessed by the one-way ANOVA followed by a Tukey’s *post hoc* comparison. Bold indicates significance (*p* < 0.05). a; cluster 1 *vs* cluster 2 (p<0.05). b; cluster 2 *vs* cluster 3 (p<0.05). c; cluster 1 *vs* cluster 3 (p<0.05).

## Discussion

This study demonstrates that aging significantly increases susceptibility to pulmonary MAC infection in mice, primarily through downregulation of the Nrf2-SLPI axis. In old mice, Nrf2 activity and *SLPI* mRNA expression were significantly decreased, resulting in higher bacterial loads and worse survival outcomes. The high susceptibility of old mice to MAC infection is consistent with previous reports (6). In the present study, SLPI was shown to have antibacterial activity against MAC bacteria for the first time, and *SLPI* expression regulated by Nrf2 contributes to the control of MAC infection. Moreover, it was shown that the activation of this Nrf2-SLPI axis was attenuated with aging. Based on the results of a comprehensive gene expression analysis of whole blood cells from patients with pulmonary MAC disease, three clusters defined by *SLPI* mRNA expression and age were identified. Two groups were identified with similarities to the results of the mouse experiment. Compared with C1, C3 included older patients with lower *SLPI* expression and greater lesion spread as indicated by CT scores. In addition, Nrf2 activation was lower in C3 than in C1. These results suggest that the Nrf2-SLPI axis is also involved in the pathophysiology of MAC disease in humans.

SLPI, a protein with known antiprotease or antimicrobial properties, was identified in this study as a potential key effector against pulmonary MAC infection in mice. In humans, SLPI has been reported to be widely expressed in the respiratory tract, gastrointestinal tract, genital tract, parotid gland, breast, kidney, and skin, and secreted by epithelial cells, macrophages, granulocytes, or dendritic cells. In the respiratory tract, SLPI has been reported to be expressed in airway epithelial cells, alveolar type II epithelium, or alveolar macrophages (16, 17). SLPI was originally described as a protein that protects against excessive tissue damage caused by proteolytic enzymes. Subsequently, it was reported to suppress inflammatory responses by regulating the activity of NF-κB, to regulate NET production, and to have direct antibacterial activity against *Staphylococcus aureus*, *E. coli*, fungi, or *Mycobacterium tuberculosis* (17, 18). Though the function of SLPI against non-tuberculous mycobacterial (NTM) infection has not been reported, the present study notably showed that the direct addition of SLPI to MAC bacteria significantly reduced the number of bacteria, confirming a direct bactericidal effect of SLPI on MAC bacteria. In addition, the expression of SLPI was significantly attenuated in old mice compared with young mice after MAC infection. These results suggest that SLPI is an important host response gene in NTM infection and a potential target for therapeutic intervention.

In MAC-infected old mice, granuloma formation was impaired compared with that in MAC-infected young mice. It has been reported that the involvement of HO-1 in granuloma formation during MAC infection in the previous studies (6, 14), However, consistent with a previous report (6), no difference in HO-1 expression was observed between MAC-infected old and young mice in this study (data not shown). They showed that the HO-1 response following infection with *M. avium* was blunted in old mice compared with young mice (6). These findings suggest that HO-1 may play a limited role in age-related impairment of granuloma formation. SLPI has been reported to bind to *Mycobacterium tuberculosis* and promote its phagocytosis by macrophages (18). Taken together, these findings suggest that reduced activity of the Nrf2–SLPI axis in MAC-infected old mice leads to increased bacterial burden and impaired macrophage phagocytic capacity, resulting in impaired granuloma formation. The putative schema is shown in **Supplementary Figure 3**.

The therapeutic potential of targeting the Nrf2-SLPI axis was also demonstrated using sulforaphane, an Nrf2 activator. Nrf2 is a transcriptional regulator of cellular homeostasis and has been reported to play a protective role against infection, as well as in the antioxidant stress response (19). Previously, we reported that NRAMP1 and HO-1, both regulated by Nrf2, promoted phagolysosome fusion and granuloma formation, respectively, contributing to infection control in a mouse model of pulmonary MAC infection (14). Furthermore, Nrf2 has been reported to induce the expression of *SLPI*, since antioxidant response element (ARE) is present in the promoter region of the *SLPI* gene (11–13), and its function decreases with aging (9, 10). In the present study, Nrf2 activation during MAC infection was significantly lower in the lungs of old mice than in those of young mice. In addition, it was confirmed that *SLPI* expression during MAC infection is attenuated in Nrf2-deficient mice. Importantly, *SLPI* expression was induced in old infected mice after treatment with SFN.

Since SFN, an Nrf2 activator, has been reported to target Keap1 (20, 21), the mechanism underlying the decreased Nrf2 activity in old mice infected with MAC may include the age-related acceleration of Nrf2 degradation via Keap1. In addition, a previous study reported that the functional decline of Nrf2 with age is associated with decreased levels of positive regulators of Nrf2, such as PI3K, p62, CBP, and BRCA1, and increased levels of negative regulators of Nrf2, such as Keap1, Bach1, and Myc (22). In the present study, DEG analysis of infected lung tissues showed no significant differences in the expressions of *PI3K*, *p62*, *CBP*, *BRCA*, *Keap1*, or *Bach1* between young and old mice. In contrast, *Myc* gene expression was significantly upregulated in the lungs of infected old mice compared with infected young mice and uninfected old mice (**Supplementary Table 2**). Myc has been shown to form a complex with Nrf2, inhibit Nrf2 binding to the ARE promoter region, and shorten the half-life of Nrf2 (23). Further studies are needed to elucidate the relationship between aging and the decrease in Nrf2 activity in pulmonary MAC disease.

In a cluster analysis of 100 patients with pulmonary MAC disease, a distinct group (C2) was identified in addition to two groups (C1, C3), which resembled the experimental results observed in mice. Interestingly, C2 included a higher proportion of elderly patients, who had higher *SLPI* expression and greater disease severity. This group also had higher CRP levels than C1 and C3, and significantly higher BACScrp values than C1. In addition, results from DEG analysis and the immune cell ratio strongly indicated prominent involvement of neutrophils in C2 compared with the other two groups. SLPI, a protease inhibitor, has the ability to inhibit neutrophil elastase released by activated neutrophils. Neutrophil inflammation and neutrophil elastase are known to play a role in the pathogenesis of NTM infection, including bronchiectasis and cavitation (24, 25). Based on these findings, we hypothesized that the increased expression of *SLPI* in C2 reflects a response to excessive neutrophilic inflammation. Given that pulmonary NTM disease has been reported to be a multifactorial condition (2, 26), the presence of C2 suggested the existence of a population characterized by a molecular pathogenesis distinct from the Nrf2-SLPI axis.

Although the present findings underscore the importance of the Nrf2-SLPI axis, some limitations should be noted. First, *SLPI* expression in whole blood cells from patients with pulmonary MAC disease may not necessarily accurately reflect gene or protein expression in infected lung tissue. Second, although mouse models provide valuable insights, interspecies differences may limit the generalizability of the results to humans. Finally, although the importance of the Nrf2–SLPI axis was demonstrated in the mouse model, these findings cannot be fully extrapolated to human pulmonary MAC disease, further highlighting the heterogeneity of this disease. Further studies are needed to determine whether the Nrf2-SLPI axis is involved in MAC bactericidal activity in human pulmonary MAC disease.

In conclusion, this study identified the Nrf2-SLPI axis as a critical mediator of age-related susceptibility to pulmonary MAC disease. Targeting this axis may be a promising therapeutic approach to improve the prognosis of elderly patients. Future studies should focus on validating these findings in larger cohorts and exploring additional mechanisms contributing to Nrf2-SLPI axis dysfunction, as well as the potential for new therapeutic strategies using Nrf2 activators or SLPI protein.

## Data Availability

The datasets presented in this study can be found in online repositories. The names of the repository/repositories and accession number(s) can be found in the article/**Supplementary Material**.

## Supplementary material

Supplementary Figure 1 Comparative analysis of Nrf2 activation between each cluster using upstream regulator analysis. This figure is a heat map based on the activation z-score, a value that predicts whether an upstream regulator is activated or inhibited. There is no bias in Nrf2 activation between C1 and C2 or between C2 and C3. However, it predicts that Nrf2 activation is reduced in C3 compared with C1.

Supplementary Figure 2 Proportion of immune cells in each cluster. The estimated proportion of immune cells in each cluster based on whole blood gene expression profiles using CIBERSORTx.

Supplementary Figure 3 Schematic representation of the role of the Nrf2-SLPI axis in elderly patients with pulmonary MAC disease. In young mice, infection with MAC bacteria activates Nrf2 to induce the expression of SLPI genes in lung tissue macrophages. SLPI directly kills MAC bacteria and is involved in infection control. In macrophages from old mice, the nuclear translocation of Nrf2 is inhibited, and expression of SLPI is decreased, making them unable to control MAC infection. Thus, Nrf2-regulated SLPI may be an important host factor in pulmonary MAC disease.

Supplementary Table 2 See attached Excel file. Differentially expressed genes between each mice group.

Supplementary Table 3 See attached Excel file. Differentially expressed genes between each cluster in 100 patients with pulmonary MAC disease.

Supplementary Table 4 See attached Excel file. All significantly enriched results of upstream analysis using IPA (p value threshold ≤0.05 using Fisher’s exact test) between each cluster.
