# Supplementary figures and images for: The Nrf2-SLPI axis in aging and its role in the pathophysiology of pulmonary Mycobacterium avium complex disease
Source: Front Immunol. 2026 Feb 25;17:1733057. doi: 10.3389/fimmu.2026.1733057 (PMC12975742; doi:10.3389/fimmu.2026.1733057)

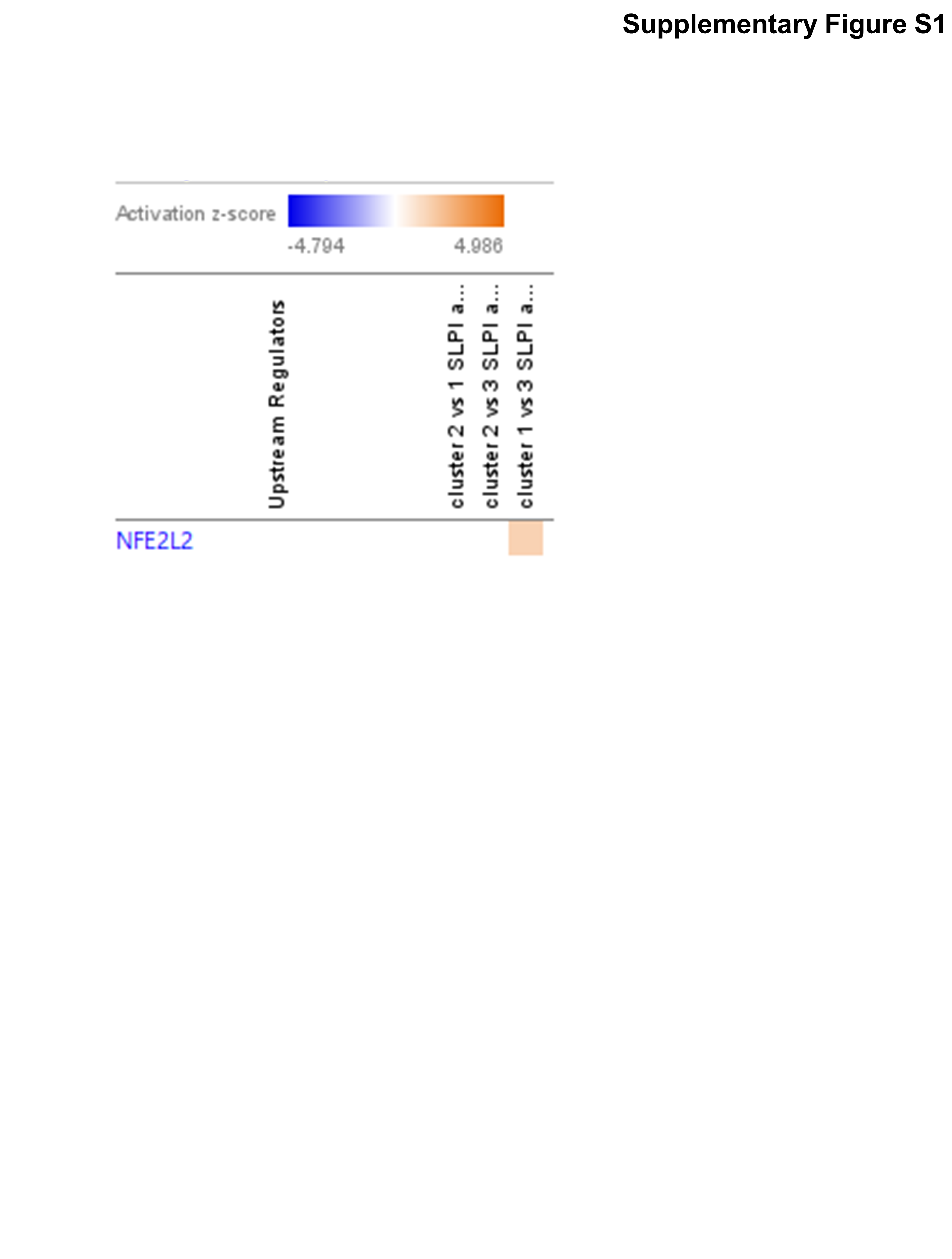

Supplement: Supplementary Figure 1 — Comparative analysis of Nrf2 activation between each cluster using upstream regulator analysis. This figure is a heat map based on the activation z-score, a value that predicts whether an upstream regulator is activated or inhibited. There is no bias in Nrf2 activation between C1 and C2 or between C2 and C3. However, it predicts that Nrf2 activation is reduced in C3 compared with C1. [file Image1.tif]

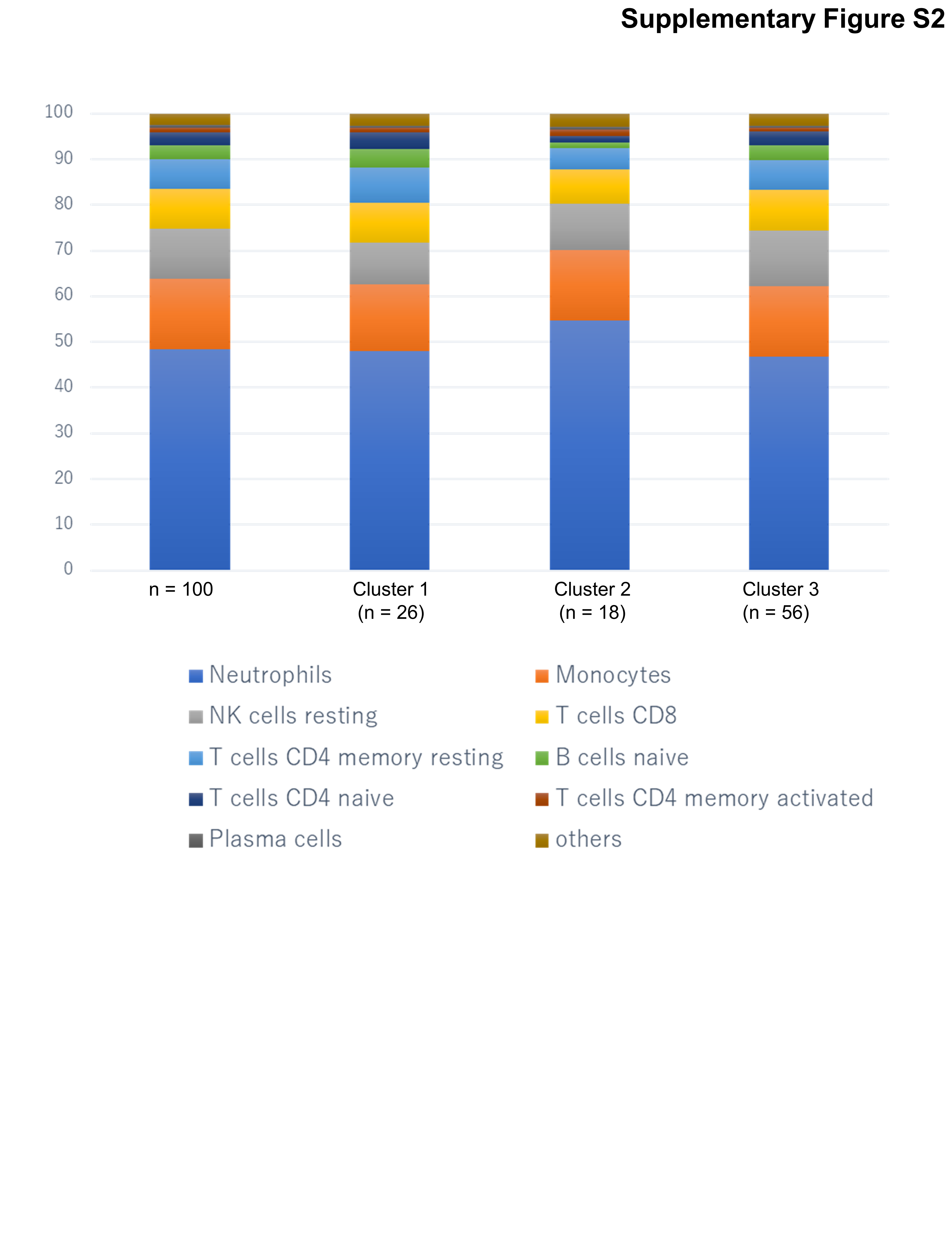

Supplement: Supplementary Figure 2 — Proportion of immune cells in each cluster. The estimated proportion of immune cells in each cluster based on whole blood gene expression profiles using CIBERSORTx. [file Image2.tif]

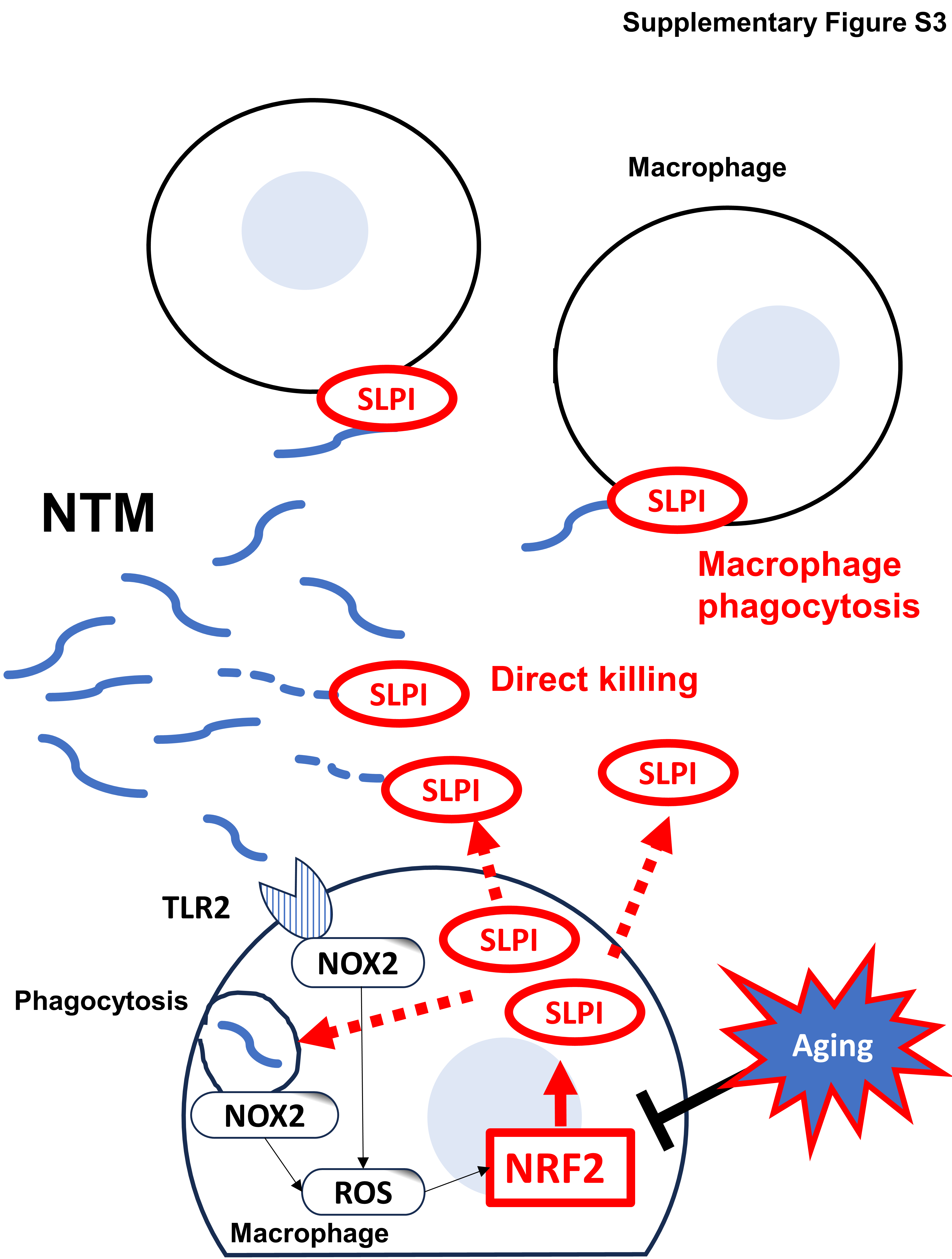

Supplement: Supplementary Figure 3 — Schematic representation of the role of the Nrf2-SLPI axis in elderly patients with pulmonary MAC disease. In young mice, infection with MAC bacteria activates Nrf2 to induce the expression of SLPI genes in lung tissue macrophages. SLPI directly kills MAC bacteria and is involved in infection control. In macrophages from old mice, the nuclear translocation of Nrf2 is inhibited, and expression of SLPI is decreased, making them unable to control MAC infection. Thus, Nrf2-regulated SLPI may be an important host factor in pulmonary MAC disease. [file Image3.tif]
