# Supplementary material for: The Nrf2-SLPI axis in aging and its role in the pathophysiology of pulmonary Mycobacterium avium complex disease
Source: Front Immunol. 2026 Feb 25;17:1733057. doi: 10.3389/fimmu.2026.1733057 (PMC12975742; doi:10.3389/fimmu.2026.1733057)
Supplement: Supplementary file 4 [file Table1.docx]

**Table S1.** **Primers used for RT-PCR**

| Primer Target | Sequence |
| --- | --- |
| GAPDH | 5’-AGG TCG GTG TGA ACG GAT TTG-3’ (forward) |
|  | 5’-TGT AGA CCA TGT AGT TGA GGT CA-3’ (reverse) |
| SLPI | 5’-GGC CTT TTA CCT TTC ACG GTG-3’ (forward) |
|  | 5’-TAC GGC ATT GTG GCT TCT CAA-3’ (reverse) |
| Nramp1 | 5’-GCA GGC CCA GTT ATG GCT C-3’ (forward) |
|  | 5’-CAG GCT GAA TGT ACC CTG GTC-3’ (reverse) |
| HO-1 | 5’-AAG CCG AGA ATG CTG AGT TCA-3’ (forward) |
|  | 5’-GCC GTG TAG ATA TGG TAC AAG GA-3’ (reverse) |
